# The Abscopal Effect in the Era of Checkpoint Inhibitors

**DOI:** 10.3390/ijms22137204

**Published:** 2021-07-04

**Authors:** Ondřej Kodet, Kristýna Němejcova, Karolína Strnadová, Andrea Havlínová, Pavel Dundr, Ivana Krajsová, Jiří Štork, Karel Smetana, Lukáš Lacina

**Affiliations:** 1 Department of Dermatovenereology, First Faculty of Medicine and General University Hospital, Charles University, 128 00 Prague, Czech Republic; andrea.havlinova@vfn.cz (A.H.); ivana.krajsova@vfn.cz (I.K.); jiri.stork@lf1.cuni.cz (J.Š.); 2 Institute of Anatomy, First Faculty of Medicine, Charles University, 128 00 Prague, Czech Republic; karolina.strnadova5@gmail.com (K.S.); karel.smetana@lf1.cuni.cz (K.S.J.); 3 Biotechnology and Biomedicine Center, Academy of Science and Charles University, 252 50 Vestec, Czech Republic; 4 Institute of Pathology, First Faculty of Medicine and General University Hospital, Charles University, 128 00 Prague, Czech Republic; Kristyna.Nemejcova@vfn.cz (K.N.); pavel.dundr@vfn.cz (P.D.)

**Keywords:** abscopal effect, cryotherapy, anti-CTLA-4, melanoma, tumor neoantigens, wound healing

## Abstract

Therapy targeting immune checkpoints represents an integral part of the treatment for patients suffering from advanced melanoma. However, the mechanisms of resistance are responsible for a lower therapeutic outcome than expected. Concerning melanoma, insufficient stimulation of the immune system by tumour neoantigens is a likely explanation. As shown previously, radiotherapy is a known option for increasing the production of tumour neoantigens and their release into the microenvironment. Consequently, neoantigens could be recognized by antigen presenting cells (APCs) and subjected to effector T lymphocytes. Enhancing the immune reaction can trigger the therapeutic response also at distant metastases, a phenomenon known as an abscopal effect (from “ab scopus”, that is, away from the target). To illustrate this, we present the case of a 78-year old male treated by anti-CTLA-4/ipilimumab for metastatic melanoma. The patient received the standard four doses of ipilimumab administered every three weeks. However, the control CT scans detected disease progression in the form of axillary lymph nodes metastasis and liver metastasis two months after ipilimumab. At this stage, palliative cryotherapy of the skin metastases was initiated to alleviate the tumour burden. Surprisingly, the effect of cryotherapy was also observed in untreated metastases and deep subcutaneous metastases on the back. Moreover, we observed the disease remission of axillary lymph nodes and liver metastasis two months after the cryotherapy. The rarity of the abscopal effect suggests that even primed anti-tumour CD8^+^ T cells cannot overcome the tumour microenvironment’s suppressive effect and execute immune clearance. However, the biological mechanism underlying this phenomenon is yet to be elucidated. The elicitation of a systemic response by cryotherapy with documented abscopal effect was rarely reported, although the immune response induction is presumably similar to a radiotherapy-induced one. The report is a combination case study and review of the abscopal effect in melanoma treated with checkpoint inhibitors.

## 1. Introduction

Immune checkpoint inhibitors represent a milestone in the immunotherapy of cancer, including melanoma. The monoclonal antibody targeting CTLA-4 (ipilimumab) was the pioneer checkpoint inhibitor on the market, achieving a significant improvement in overall survival (OS) in metastatic melanoma patients. Despite this success, the therapeutic response observed in patients treated by ipilimumab is only about 10% [1]. However, the most commonly used checkpoint inhibitors nowadays, anti-PD-1 (nivolumab, pembrolizumab), have a significantly higher response (up to 45%). The best response was observed with combination therapy of anti-CTLA-4 and anti-PD-1 (ipilimumab, nivolumab, as high as 58% objective response rate, ORR). Not surprisingly, this combination therapy is associated with higher serious side effects of the grades 3 and 4 [2]. Despite these advances in the treatment of metastatic melanoma, many patients do not benefit from immunotherapy.

The mechanisms of resistance to immunotherapy in melanoma are numerous and reflect the complexity of tumour cell interactions with the immune system. Not all of these interactions are fully documented and understood. The principal mechanisms of immunotherapy resistance include:

- insufficient production and release of neoantigens by the tumour (presumably due to a low mutation load) with consequent insufficient stimulation of immune cells by these neoantigens [3,4];
- insufficient tumour infiltration by tumour infiltrating lymphocytes (TILs) resulting in immunologically “cold” or “deserted” tumours [5];
- absence of PD-1-expressing T cells and only transient infiltration of PD-L1-expressing tumour-associated macrophages (TAMs) in metastasis, which might be documented in biopsies at the beginning of therapy [6];
- presence of an innate transcriptional “signature” of anti-PD-1 resistance (IPRES, innate PD-1 RESistance) [7];
- insufficient signalling of interferon γ [7,8,9,10,11].

Low stimulation of the immune system by tumour-specific neoantigens is indeed one of the critical factors responsible for immunotherapy resistance [12]. The entire concept of the immune system function is based on precise discrimination and response to antigenic stimuli. The immune system can either recognize and tolerate, or destroy if necessary. T-lymphocytes sensitively and specifically differentiate tumour cells from the cells of healthy tissues and thus execute efficient immune surveillance. Melanoma is one of the tumours with the highest mutation burden in humans [13]. This should theoretically increase the possibility of eliciting an immune response [12]. Sadly, some tumours (including melanoma) do not sufficiently produce neoantigens or do not adequately present these neoantigens to the immune system and, hence, cannot be recognized as foreign [14].

The abscopal effect is based on these principles of immune system surveillance [15]. The abscopal effect has been hypothesized as a concept in metastatic cancer treatment since the 1950s. The term “abscopal” (from Latin, “ab”—away from, “scopus”—target) was used first (in 1953) to describe the observed therapeutic effect of ionizing radiation in the anatomic site, which was not irradiated [16]. In later years, the abscopal effect was observed repeatedly after radiotherapy. In this context, the tumour area (e.g., regional lymph nodes basin) was irradiated. This treatment was followed by surprising regression of metastasis also in distant non-irradiated regions. Since the earliest observations of this phenomenon, it was hypothesized that local radiation triggered a systemic immune response [17,18,19]. The abscopal effect has been reported in several malignancies, including malignant melanoma or non-small cell lung cancer [17].

The abscopal effect has also been observed after various other types of local treatments such as electroporation or intra-tumoral injection of therapeutics [20,21]. However, the abscopal effect associated with cryotherapy was only scarcely described, and the available data are limited [22].

Mechanistically, the disintegration of tumour cells due to local treatment can release tumour neoantigens. This prerequisite could consequently result in the stimulation of the immune system. Recognition of the tumour-specific neoantigens, their intake and presentation are mediated by dendritic cells (DCs).

These antigen-presenting cells play a crucial role in the initiation and regulation of the innate and adaptive immunity. DCs induce a specific anti-tumour immune response after processing tumour-specific neoantigens [14,23]. The antigens are then presented to CD8^+^ T cells by MHC molecules class I and to CD4^+^ T cells by MHC molecules class II. Antigen presentation efficiently performed by mature DCs also contributes to the cytotoxic immune response by activating NK cells [24,25].

The abscopal effect was also studied in mouse models where localized radiation was used. In the mouse melanoma model, CD8^+^ T cells were required to reduce melanoma burden following radiotherapy, and immunotherapy enhanced the response [26]. The abscopal effect has also been observed in wild-type mice treated with a combination of radiotherapy and Flt3-L, a growth factor that stimulates the production of DCs [27]. This study supported the importance of DCs in the induction of the anti-tumour immune response.

Most recently, the abscopal effect attracted the attention of researchers due to the possibilities of combining checkpoint inhibitors and radiotherapy. In a clinical study (MASTERKEY 256) using a combination of radiotherapy and ipilimumab, the abscopal effect was also observed. Later, it was also observed with the combination of talimogene laherparepvec (T-VEC) oncolytic virus vaccine and pembrolizumab [28].

The intratumoral administration of therapeutic viral particles leads to oncolysis; however, it also attracts immune cells to the treated metastatic lesion with an increase of CD8^+^ T cell infiltration in the tumour stroma [29]. Furthermore, reactive expression of PD-L1 in the tumour microenvironment promotes concomitant blockade of PD-1. After combined therapy, tumour antigen-specific CD8^+^ T cells that were fully stimulated in the injected lesion can traffic and infiltrate distant metastatic lesions to exert systemic anti-tumour activity, thereby reversing primary resistance to PD-1 blockade therapy [28].

**Figure 1:**
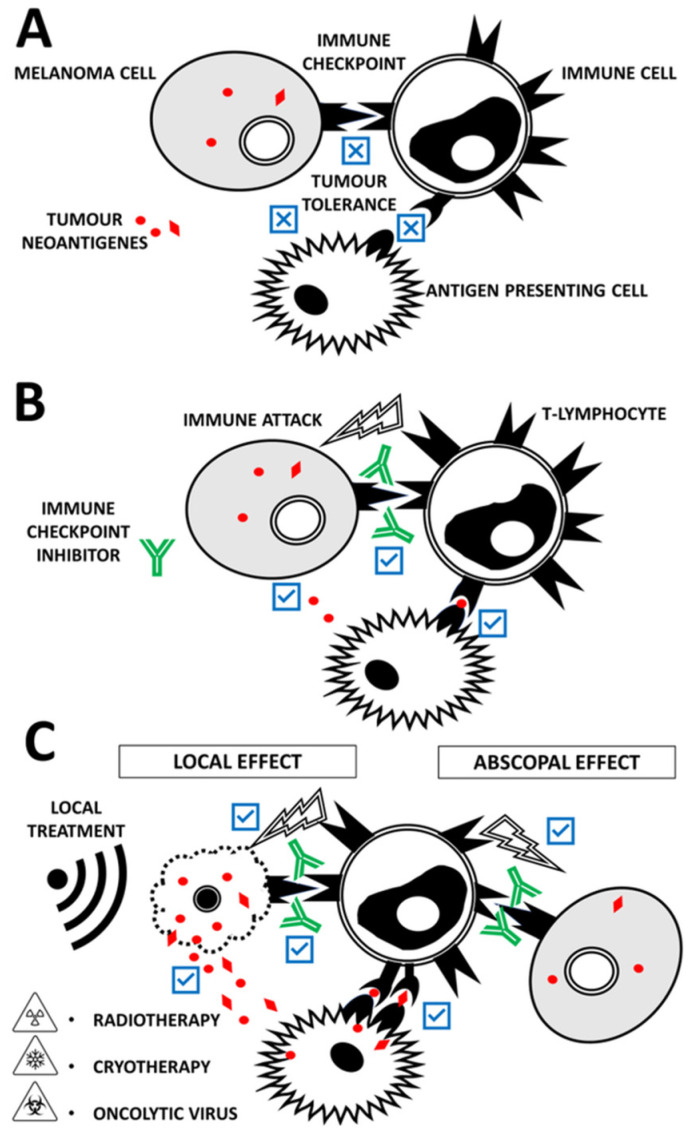
(**A**) Insufficient production and release of neoantigens by the melanoma cells lead to insufficient stimulation of effector immune cells (if present) by antigen-presenting cells. Tumour cells also express negative regulators of T-cell function. The most prominent druggable checkpoint molecules are CTLA-4 and PD-1/PD-L1 (not differentiated); all of them have been associated with immune evasion in cancer. Thus, tumour tolerance can be established. These mechanisms allow cancer cells to avoid destruction by the immune attack. (**B**) Administration of immune checkpoint inhibitors such as anti-CTLA4, anti-PD1 and anti-PD-L1 has remarkable success in enhancing the effector anti-tumour response and triggering the immune attack. However, not all patients respond completely to checkpoint inhibitors. Also, tumour cells can gradually develop mechanisms to avoid immune attack. (**C**) Checkpoint inhibitor therapeutical outcome can be improved in combination with radiotherapy, cryotherapy or oncolytic viruses. All these treatment modalities act locally. Tumour cell damage increases the availability of tumour neoantigens for antigen-presenting cells and effector immune cells. However, immune system activation and thus a therapeutic effect can be observed in the anatomic site, which was not affected by local treatment. This is known as the abscopal effect.

We aimed to review the abscopal effect (Figure 1) and its immunological and molecular mechanisms in the context of current immune checkpoint inhibitor therapy. In this light, we demonstrate clinically the abscopal effect observed at our department in a melanoma patient.

## 2. Case Report

### 2.1. Patient

**Figure 2:**
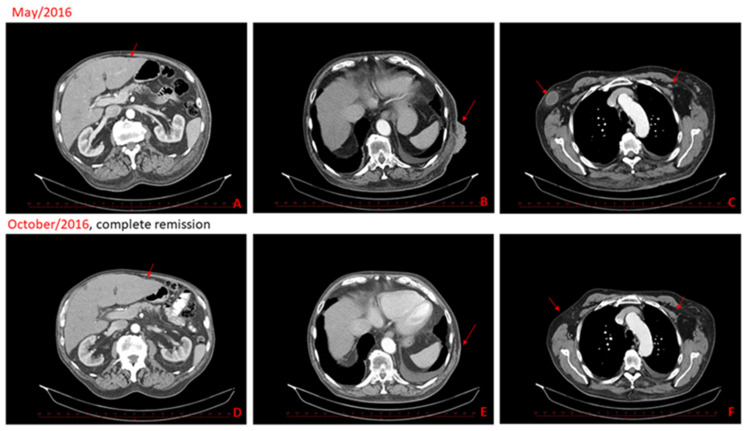
CT scans from May 2016 show progressive disease, new liver metastasis (**A**), skin and subcutaneous metastasis (**B**), left subclavicular node metastasis and right node axillary metastasis (**C**). CT scans in the second-line show complete remission of the metastatic disease, two months after cryotherapy and half a year after the last dose of ipilimumab (**D**–**F**).

**Figure 3:**
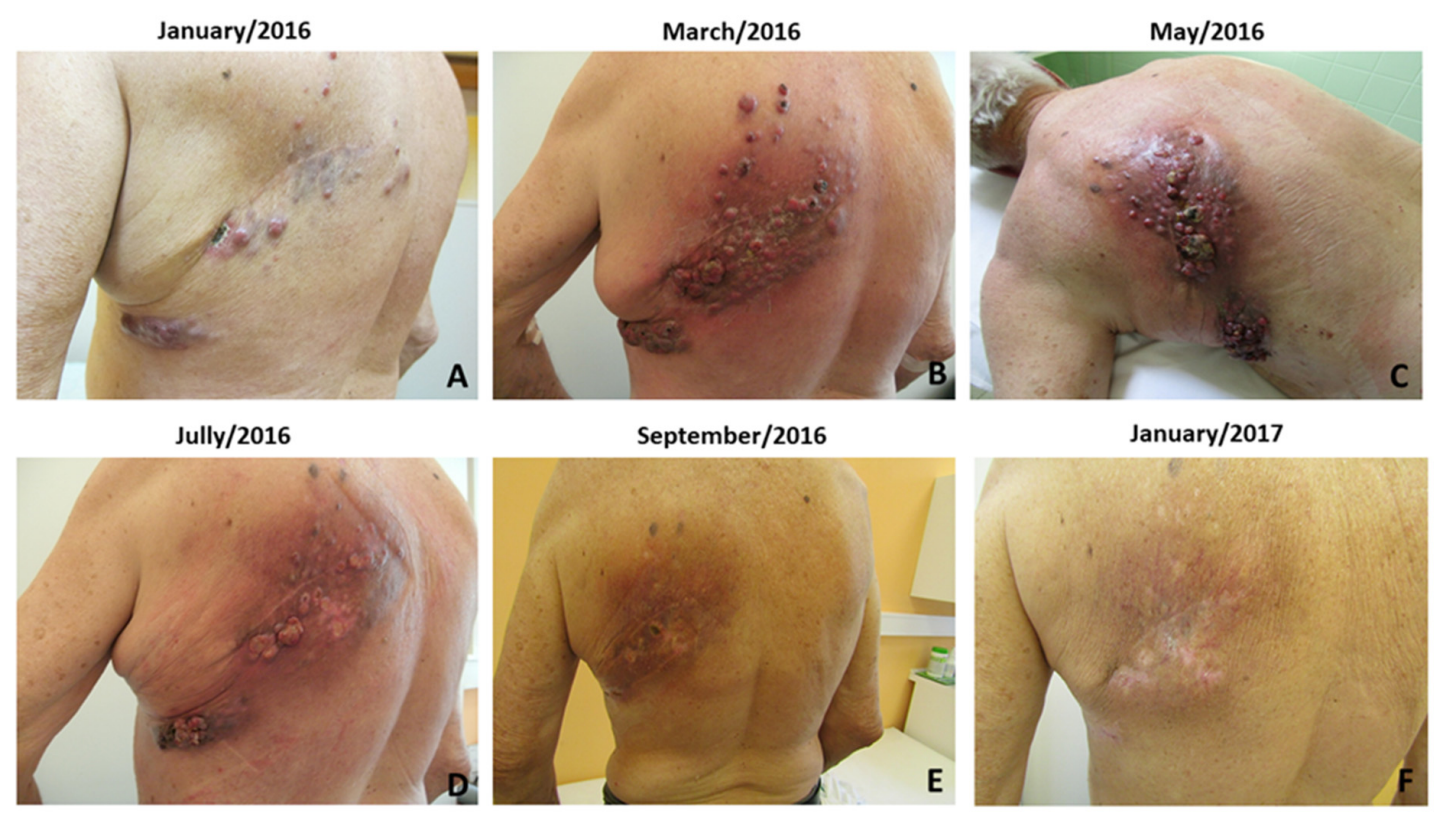
Clinical features at the beginning of ipilimumab therapy (**A**), after ipilimumab therapy cessation (**B**) and during perioperative preparation before cryotherapy (**C**). In the second row, we show clinical features during regression, before the 2nd cryotherapy (**D**), where partial regression was present, and the patient reported significant relief. Next, clinical pictures show practically healed skin lesions two months after cryotherapy (**E**), and already fully healed lesions on the back, where only scarring changes are visible, half a year after cryotherapy (**F**).

A 78-year old Caucasian male suffering from metastatic melanoma was referred to our centre for treatment. Primary cutaneous melanoma (Breslow 1.5 mm, without ulceration, BRAFwt) was widely excised from the patient**’**s dorsal skin in 2009. Six years later, we detected disease progression with numerous cutaneous and subcutaneous metastases at the original scar on back in 2015. The patient received standard ipilimumab (anti-CTLA-4) treatment (four doses, 3 mg/kg, every three weeks). Unfortunately, further disease progression was confirmed on a CT scan with new axillary lymph nodes metastasis and liver metastasis two months after the last ipilimumab dose administration (Figure 2). The cutaneous metastases were also notably progressing (Figure 3). Therefore, we initiated at this time palliative cryotherapy of accessible cutaneous metastases to reduce the tumour burden and alleviated discomfort associated with lesion oozing and haemorrhage.

### 2.2. Patient Follow Up

Contrary to the situation before the cryotherapy, the cutaneous, liver and axillary lymph node metastases were not detected on the control CT scan (Figure 2D,F) two months after cryotherapy (approximately fourth months after the last ipilimumab administration). An excellent clinical response was apparent as early as two months after cryotherapy, where almost healed sites of cutaneous metastases were observed (Figure 3A–F). Excellent clinical response and fully healed skin lesions were documented during a regular follow-up visit six months after cryotherapy (Figure 3F). The complete response was maintained for 13 months after cryotherapy and 15 months after the last application of ipilimumab. The patient ceased suddenly due to myocardial infarction 15 months after cryotherapy, aged 79, without any clinical signs of melanoma recurrence. The autopsy also did not show any recurrence of melanoma.

### 2.3. Skin Biopsy and Immunohistochemistry

Skin biopsies were obtained at three time points. First, before administering the first ipilimumab treatment dose (January 2016), second—after the fourth dose of ipilimumab (March 2016) and the third was taken at the beginning of the second cryosurgery (July 2016). The primary objective was the monitoring of TILs.

In the first biopsy, routine staining H&E showed minimal intratumoral infiltration of TILs and irregular atypical tumour melanocytes before ipilimumab initiation (not shown). The second biopsy (after completing ipilimumab therapy) revealed a clear difference in the tumour melanocytes’ morphology. We observed smaller, relatively regular and uniform tumour cells (not shown). In the third biopsy (after initial cryosurgery), massive necrosis and marked infiltration of immune cells were seen in the tumour centre (not shown).

**Figure 4:**
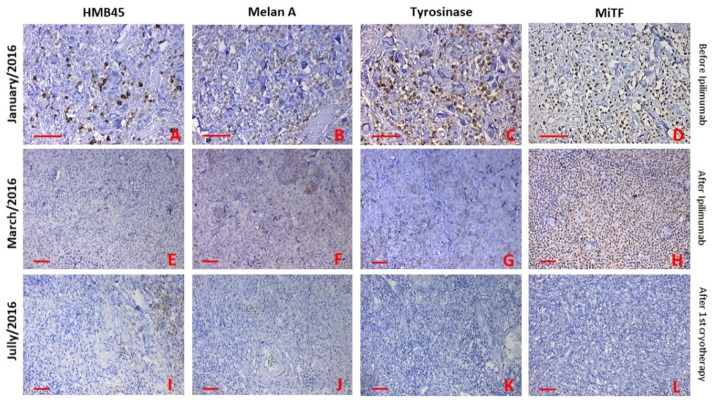
Immunohistochemical staining was performed to compare the phenotype of skin metastases, before initiation of ipilimumab therapy, after the last dose and after the first cryotherapy. The expression of all markers (HMB45, Melan A, Tyrosinase and MiTF) were present before initiation of ipilimumab therapy (**A**–**D**). MiTF and tyrosinase were only positive after the last dose of ipilimumab (**G**,**H**). HMB45 and Melan A were negative (**E**,**F**). Only HMB45 was positive in some melanoma cells at the edge of the necrotic tissue (**I**). Other markers were negative (**J**–**L**). Bar 50 μm.

Immunohistochemistry showed positivity for typical melanocytic/melanoma markers such as HMB45, MelanA/MART-1, tyrosinase and MiTF in the first biopsy (before ipilimumab, Figure 4A–D). In the second biopsy, only MiTF was positive, and other markers were negative (after ipilimumab, Figure 4E–H). The third biopsy was positive only for HMB45 in the tumour’s vital periphery (after cryotherapy Figure 4I–L).

**Figure 5:**
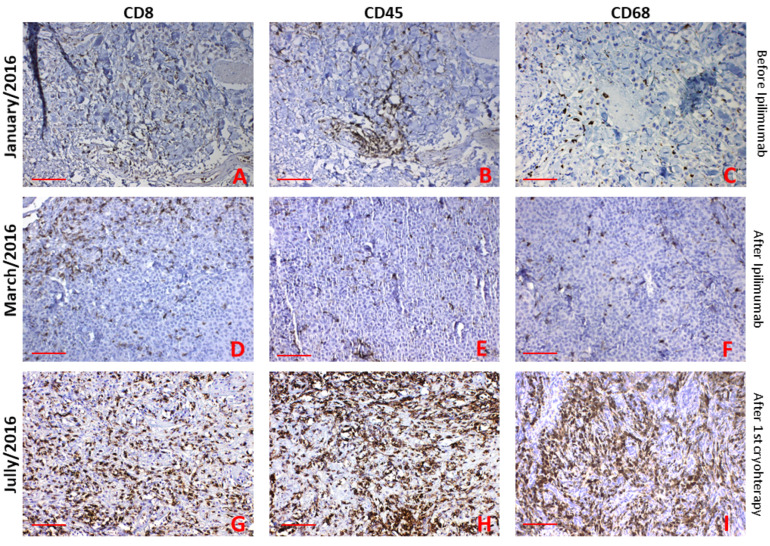
Immunohistochemical staining of TILs was also made. TILs were detected as only small perivascular infiltrate CD8 and CD45 positive cells before ipilimumab treatment (**A**,**B**). CD68 positive macrophages were seen as small diffuse infiltrate (**C**). CD8 positive T cells were observed at the edge of the tumour (**D**), and small diffuse infiltrates of CD45 and CD68 positive cells were observed after ipilimumab treatment (**E**,**F**). Significant and massive infiltrates of CD8, CD45 T cells and CD68 positive macrophages were observed in the skin metastasis after the first cryotherapy (**G-I**). Bar 50 μm.

In the first biopsy, immunohistochemistry demonstrated biopsy diffuse very small infiltrate of CD8^+^ T-lymphocytes (TILs) and CD68^+^ macrophages and perivascular infiltrate of CD45^+^ T-lymphocytes (Figure 5A–C). In the second biopsy (after ipilimumab treatment) there was small infiltrate of CD8^+^ T-lymphocytes at the periphery of the skin metastasis. This biopsy coincided with clinical progression of the disease. Virtually no infiltrate of CD45^+^ T-lymphocytes, and no CD68^+^ macrophages were seen (Figure 5D,F).

In the biopsy one month after initial cryotherapy, we saw significantly increasing infiltration with CD8^+^ and CD45^+^ T-lymphocytes and CD68^+^ macrophages (Figure 5G–I).

### 2.4. Blood Test, Tumour Markers

**Figure 6:**
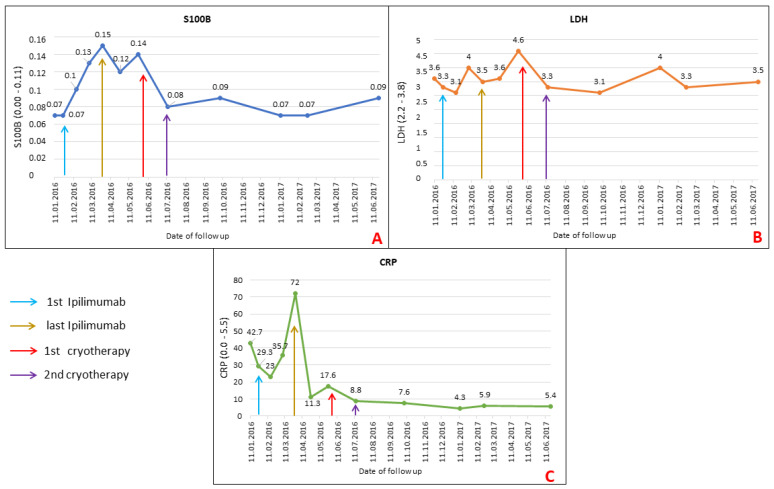
This figure shows the serological analysis of S100B, LDH and CRP during the patient’s follow up. **Graph A** shows the elevation of S100B during clinical worsening and progression of metastatic disease at the ipilimumab therapy and the normalization of S100B values after cryotherapy. Similar results and values were observed for LDH (**Graph B**). In the case of CRP, significantly lower values were observed after completion of ipilimumab therapy, despite significant clinical progression (**Graph C**).

Routinely used serological tumour markers such as S100B, LDH and CRP were used to follow the disease progression. The actual values of these biomarkers are presented in Figure 6.

S100B and LDH show elevation above the normal value at the end of ipilimumab therapy during clinical progression and decline to normal after cryotherapy and subsequent regular follow-ups (Figure 6A,B).

The value of CRP increased during ipilimumab therapy and, surprisingly, decreased after the end of ipilimumab treatment before cryotherapy (Figure 6C). However, there was an inflammatory response in the vicinity of skin metastases.

Despite the relatively good clinical and laboratory status before initiating ipilimumab therapy, the neutrophil to lymphocyte ratio (NLR) was unfavourable. The NLR was 3.69 (Neutrophils abs.: 4.99 [2.00–7.00]/Lymphocytes abs.: 1.35 [0.80–4.00]) before initiating ipilimumab therapy, and NRL was 1.74 (Neutrophils abs.: 3.66/Lymphocytes abs.: 2.10) (for details and reference values see Table 1) at the end of this therapy. Although it is not a factor that excludes checkpoint inhibitor therapy, NLR may be a helpful biomarker of the overall therapeutic response and time to progression-free survival (PFS) or overall survival (OS) [30].

## 3. Discussion

Ipilimumab has shown significant improvement in OS in metastatic and advanced melanoma in two randomized, phase 3 clinical trials with response rates of 10–15% [1,31]. Although the figure is relatively low, it was a pioneer drug and undisputed promise for a new melanoma immunotherapy trend. In our case, we also observed progression on CT scans and organ metastasis early after the last ipilimumab dose administration.

In later years, several studies have sought to increase the effect of ipilimumab. Recently, the most effective is the combination of two checkpoint inhibitors, ipilimumab and anti-PD-1 inhibitor nivolumab [2].

Further desirable potentiation of checkpoint inhibitors therapy can also be achieved by radiotherapy [19]. Unfortunately, only some patients can benefit from this stimulation of the immune system with an anti-tumour effect. Due to disease dissemination, we did not choose radiotherapy in our case.

The systemic anti-tumour effect induced by focal radiotherapy, generally referred to as the abscopal effect, cannot be easily predicted by any biomarker. Chandra and co-workers described the benefit of combined radiotherapy and ipilimumab with a median OS of 28 months, compared with a median OS of 11.4 months in the clinical trial with ipilimumab [32]. Grimaldi and co-workers describe the abscopal effect in 21 patients who progressed after ipilimumab and received radiotherapy. In 11 patients (52%), the abscopal effect was observed, including nine who had a partial response (PR) and two that had stable disease (SD). The median overall survival (OS) for patients with the abscopal effect was 22.4 months vs. 8.3 months for patients who did not experience this effect [33].

However, the optimal induction of anti-tumour immunity and radiotherapy’s abscopal effect is not established in clinical practice. Recently, studies have suggested that adequate stimulation of the immune system can be achieved by a single dose of 15 Gy fraction. It is a sub-curative dose, which can slow the tumour growth as a dynamic balance between tumour proliferation and CD8^+^ T cell-mediated tumour-cell apoptosis [34,35]. Another preclinical study demonstrated the best induction of anti-tumour immunity using hypofractionation (three times 8 Gy) radiotherapy [35,36].

To summarise, the median radiotherapy dose used in patients after ipilimumab was 26 Gy (range: 8–68 Gy), and the median fraction size was 4 Gy (range: 1.8–25 Gy). The median time between radiotherapy and ipilimumab was one month [19]. The effect of stereotactic ablative body radiotherapy (SABR) has also been published to improve anti-tumour activity stimulation and control disease-free survival in multiple clinical studies [37,38].

Some tumours lack the appropriate inflammatory cytokines and chemokines to attract immune cells, such as DCs, macrophages and cytotoxic T cells, to the tumour site. Indeed, we have also observed this phenomenon in the initial biopsy. On the other hand, the expression of immunosuppressive ligands and death ligands inhibits T cells’ function and activation of the anti-tumour effect [39]. The downregulation of adhesion molecules, such as vascular cell adhesion molecule 1 (VCAM1) and intercellular adhesion molecule 1 (ICAM1), in the tumour microenvironment can inhibit T cell migration to the tumour site. On the other hand, these molecules’ expression promotes cell invasion and metastasis and is associated with a worse prognosis in melanoma patients [40,41].

Radiotherapy can promote the release of tumour neoantigens and potentially lead to the stimulation of immune responses with an anti-tumour effect. Reits and co-workers observed that radiotherapy at doses 10–26 Gy could enhance MHC-I expression in vitro and in vivo studies. This fact increased the presentation of tumour antigens and stimulated T cell attack to the tumour site [42]. The next anti-tumour activity of radiotherapy can occur by introducing Fas ligands expression and ICAM-1 molecule on tumour cells, making tumour cells more sensitive to T cell-mediated lysis [43].

The change in the tumour microenvironment by radiotherapy is relatively fundamental. Localized radiotherapy induces a burst release of cytokines and chemokines, giving rise to an inflammatory tumour microenvironment. The main factor is the strengthening of interferons’ expression (IFNs), crucial for anti-tumour immune response. Type I IFN is essential for the activation and function of DCs and T cells, which, in turn, is responsible for the release of IFN-γ and tumour control. Inhibition of expression IFN-γ is one of many factors responsible for resistance to immunotherapy by checkpoint inhibitors [44,45].

Radiotherapy induces cell apoptosis called immunogenic cell death (ICD). This type of apoptosis responds to the release of tumour neoantigens for stimulation of antigen-presenting cells (APCs). It is the stimulation of these cells that is responsible for the possible systemic immune response and the induction of the abscopal effect [46]. Calreticulin is a molecule transported from the endoplasmatic reticulum to the cell surface due to radiotherapy’s stress factors. This expression may by the signal for APCs, resp. DCs for phagocytosis of damaged cells by radiotherapy [47]. Another molecule is adenosine triphosphate (ATP), which can attract monocytes and DCs to tumours by a purinergic receptor P2 × 7-dependent pathway and promote the secretion of pro-inflammatory cytokines such as IL-1β and IL-18 [48]. During activation of anti-tumour immunity and pro-inflammatory microenvironment, Toll-like receptors play a significant role by binding to a molecule such as HMGB1 (high-mobility group box protein 1). This expression is associated with tumour cell stress after radiotherapy [49]. After stimulation of DCs with neoantigens, these cells migrate to lymph nodes to present the neoantigens to T cells by MHC molecules pathway and T cell receptors. The primary activation is the stimulation of CD8^+^ T cells, which are crucial effectors in anti-tumour immunity. However, the MHC pathway is a complex of interactions that, alone, are insufficient to lead to T cells’ activation. Subsequent activation is necessary for co-stimulatory signals, such as the expression of CD80, CD40 L, and CD28 [50]. O ne dominant negative immunomodulatory receptor is cytotoxic T lymphocyte-associated antigen 4 (CTLA-4). It can also combine with CD80 and CD86 and attenuate T cell activation. Targeted blockade of the CTLA-4 receptor allows stimulation of the immune system with a systemic anti-tumour effect after stimulation with tumour neoantigens [51]. Therefore, the combination of ipilimumab and radiotherapy (or other modalities to induce cell lysis and release neoantigens) helps potentiate the systemic anti-tumour effect and, in some cases, also the abscopal effect [17,19,33]. In the case of programmed cell death 1 (PD-1), it is expressed in T cells’ plasma membranes, DCs and NK cells. PD-1 interacted with ligands, PD-L1 and PD-L2, which are ideally expressed in tumour cells. In some tumours, such as non-small cell lung cancers, PD-L1 expression is essential for PD-1 inhibitors therapy [52,53]. PD-L1 is not routinely investigated in melanoma, and even patients without expression may have an excellent therapeutic response to PD-1 inhibitors, however, worse than in patients with fully expressed expression [2]. The upregulation of PD-L1 can be observed in mouse breast cancer and colon adenocarcinoma tumour mouse models [54]. In this vein, the combination of checkpoint inhibition by PD-1/PD-L1 can be enhanced by radiotherapy to achieve a systemic anti-tumour effect. Park and co-workers investigated the influence of PD-1 expression on the systemic anti-tumour response with abscopal effect induced by SABR in preclinical mouse melanoma and renal cell carcinoma models. SABR induces a tumour-specific immune response in the irradiated and non-irradiated (abscopal) tumours, potentiated by PD-1 blockade [35]. Tsui and co-workers describe a patient with mucosal melanoma treated with adjuvant radiotherapy (50 Gy, 20 fractions) and with palliative radiotherapy by 24 Gy in 3 fractions (0, 7 and 21. day) for neck lymph node progression of metastatic disease after pembrolizumab [55].

Cryotherapy with liquid nitrogen is a possible option to treat non-melanoma skin cancers such as basal cell carcinoma [56]. In melanoma, cryosurgery can be used as a palliative treatment of skin metastases, to a limited extent of liver metastases. It can be a helpful option during surgical extirpation or adjuvant therapy of conjunctival melanoma [57,58,59].

The direct cellular injury in cryotherapy is due to the freezing of water, leading to disruptive necrosis and water transmembrane movement. Of note, thawing is also associated with cellular damage and can be equally destructive. The mechanisms by which cryoablation kills cells are therefore multiple and complex. Mechanistically, the cooling and warming rate during cryosurgery has a significant impact on extracellular and intracellular ice formation and also osmotic changes. In our case, we have indeed observed massive necrosis and marked infiltration of immune cells in the third presented biopsy (Figure 4I–L).

The release of many tumour neoantigens induced by necrosis due to cryotherapy could be one of the main reasons for the stimulation of the immune system and the abscopal effect in our case. Our observation can support this, because we have seen significantly increasing infiltration with CD8^+^, CD45^+^ and CD68^+^ cells in tumours and their vicinity after cryotherapy (Figure 5G–I).

The attention of contemporary immunooncology is primarily focused on the aspects of the adaptive immune response following cryosurgery [60]. However, the initial immunologic consequence of cryoablation is the initiation of a purely nonspecific innate response.

Gazzaniga and co-workers observed a quick infiltrative response of polymorphonuclear leukocytes around the cryoablated tumours in a murine model in the first 24 h [61]. The neutrophilic infiltration was peaking at day three and was consequently replaced by extensive macrophage recruitment which was present after day three. Notably, the authors observed that an early intratumoral immune cell infiltration was detected only in animals receiving recombinant murine granulocyte macrophage- colony-stimulating factor 24 h after cryoablation of xenotransplanted melanoma.

Macrophages are a critically important cell type in the clearance of necrotic tissue. Moreover, macrophages are also active players in immunomodulation after cryoablation. Macrophages secrete various cytokines that can attract, activate and further orchestrate multiple other immune cells. Of note, macrophages are highly heterogeneous in these activities. In the last decades, the remarkable plasticity of macrophages in response to different environments was gradually studied, and the concept of M1/M2 polarization was broadly accepted [62]. M1 macrophages, also known as classically activated macrophages, are pro-inflammatory and have a central role in host defence against infection. On the contrary, M2 macrophages, known alternatively as activated macrophages, are associated with responses to anti-inflammatory reactions and tissue remodelling. The transformation of naïve macrophages to different phenotypes depends on the context of the tissue microenvironment. M1 and M2 macrophages represent only two terminals of the full spectrum of macrophage activation. In the context of tumour biology, another important subgroup attracted the attention of immunologists—tumour associated macrophages. These macrophages help tumour cells escape from being killed and support them in spreading to other tissues and organs [63].

In non-infectious inflammations, such as cryoablation injury, damage-associated molecular patterns (DAMPs) such as DNA and heat shock proteins (HSPs) from damaged or dying cells can stimulate Toll-like receptors of macrophages and dendritic cells, leading to the release of various cytokines, namely IL-6 as a key secreted cytokine.

It was observed that the number of local infiltrated macrophages and serum IL-6 levels peaked at day 7 and decreased at day 14 after cryoablation [64]. Gu and co-workers also suggested here that post-cryosurgery serum IL-6 increase is mainly due to infiltrating macrophages. Of note, we can confirm these CD68 positive macrophages also in the later stage (Figure 5I, one month after cryotherapy).

Takahashi and co-workers used cryosurgery in the mouse melanoma and Lewis lung carcinoma model. This study suggested that cryosurgery generates the most favourable immune-regulatory response for abscopal tumours and activation of anti-tumour immune cells, and increased secretion of pro-inflammatory cytokines such as IL-1β, IL-2, IL-6, IL-12β, IFN-γ and TNF-α. The infiltration of CD4^+^ and CD8^+^ T cells in the abscopal tumour was also significantly higher [65].

Mechanistically, cryosurgery results in an acute response of the tissue similarly to normal wounding. Following the lysis of tumour cells, tumour-derived antigens are released. Highlighting this boosting effect of cryosurgery, some authors have referred to this ability of cryotherapy to load dendritic cells and consequently present to effectors as producing an “in-vivo dendritic cell vaccine” [66]. After cryotherapy tumour antigens, the immune system’s primary stimulated with anti-CTLA-4, and anti-PD-1 resulted in a robust cytotoxic CD8^+^ T-cell response with a potential systemic effect [22].

However, the tumour microenvironment, including malignant melanoma, is very similar to that of a chronic wound [67,68]. This microenvironment has a stimulatory effect on melanoma cells growth and migration [69]. Of note, this microenvironment represents a highly dynamic system. As cryotherapy represents the acute phase of wounding, its “healing” is associated with time-limited activation of immune system in order to remove tissue debris and minimize risk of infection [70]. This also establishes a new immune equilibrium and changes the tumour promoting setting of the cancer-associated microenvironment.

We can hypothesize that the wound healing-dependent activation of immune response in cryo-ablated tumour tissue can synergize with immune checkpoint inhibition such as anti-CTLA-4/ipilimumab therapy of melanoma.

## 4. Methods and Materials

### 4.1. Cryotherapy and Sample Collection

Cryotherapy applying extremely cold cryogen (−196 °C, liquid nitrogen) to destroy metastatic tissues was performed using contact Cryocauter (KCH 450 Automat, Czech Republic). First, we made a shave excision of five bleeding exophytic metastases to achieve a more reliable cryodestruction. Every metastasis base was cryocauterised by the method of partially overlapping fields; every field was exposed twice for 30 *s* to −189 °C with tissue warming up to 37 °C in between. The photodocumentation was performed during the first cryotherapy (Figure 1C). The second cryotherapy was completed one month later using the same procedure as above. The significant reduction of skin metastasis was evident (Figure 1D). The biopsies of skin metastasis were taken before ipilimumab initiation, during progression after ipilimumab cessation, and one month after the first cryotherapy to control the effect of immunotherapy. We also made a blood test for tumour markers such as S100B protein and lactate dehydrogenase (LDH) and C-reactive protein (CRP) at each clinical examination. According to the Declaration of Helsinki, all tissue samples, blood samples, clinical photography and CT scans were obtained after attaining informed consent with the local ethical committee’s agreement.

### 4.2. Immunohistochemistry

Tissue samples were fixed for 24–48 h in 4% neutral buffered formalin at room temperature and routinely processed to paraffin blocks. Sections (3 µm thickness) were deparaffinized and rehydrated through xylene and ethanol, and PBS. Afterwards, heat-induced epitope retrieval was performed using citrate buffer (pH 6.0) in an autoclave at 120 °C for 3 min with consequent gradual cooling to room temperature over 60 min. Unspecific binding of antibodies was inhibited using Protein Block system (Dako; Agilent Technologies, Inc., Santa Clara, CA, USA; cat. no. X0909) followed by treatment with 3% hydrogen peroxide (diluted in PBS; Sigma Aldrich; Merck KGaA) for 20 min. Sections were incubated overnight at 4 °C with biotinylated primary antibodies (manufacturer-validated summary antibody information in Table 2; the manufacturer validated all employed antibodies for use in these methods). The next day, sections were extensively washed in running water and incubated with a secondary (polymer HRP-tagged) antibody. DAB (3,3′-Diaminobenzidine) chromogen was used for visualization of immunohistochemical reaction according to the standard protocol. Nuclei were counterstained with Gill’s haematoxylin and mounted in Pertex (Biotech, Prague, Czech Republic).

### 4.3. Serological Analysis and Blood Count

All serological tests and blood counts were performed during routinely scheduled examinations as recommended for patients during follow-up after immunotherapy. Blood tests were performed in an accredited university hospital laboratory (Institute of Medical Biochemistry and Laboratory Diagnostics, General University hospital and First Faculty of Medicine, Charles University, Prague, Czech Republic). The laboratory complies with national accreditation standards (ČSN, EN ISO 15189; 2013), which guarantee the accuracy of verified results. Normal value range and pathological limits were determined by the producer. S100B (Roche, Switzerland) was performed by Electro-chemiluminescence immunoassay (ECLIA) for the in vitro quantitative determination of S100 in human serum. LDH (Roche, Switzerland) was performed by International Federation of Clinical Chemistry (IFCC) methods, and CRP (Roche) was performed by nephelometry using a Dade Behring BNII instrument. Blood count, neutrophils abs. and lymphocytes abs. were performed by automatic analyzer. The serological markers used, and the examination of the blood count, physiological limits and critical values are summarized in Table 1.

## 5. Conclusions

The abscopal effect is mediated by a systemic anti-tumour immune response elicited by focal treatment and followed by regression at distant sites (Figure 1). The abscopal effect was observed mainly after radiotherapy. Recently, it is possible to use targeted oncolytic viruses (e.g., T-VEC), which promote anti-tumour immune responses that lead to the abscopal regression of distant metastases [71]. Other options for the local treatment of patients with solid tumours include intratumorally applied cytotoxic proteins, photodynamic therapy, laser therapy, electroablation and high-intensity focused ultrasonography.

The biological rationale behind the abscopal effect triggering remains unclear. However, the great success of immunotherapy achieved in melanoma recently also reignited interest in the abscopal effect. The discussed studies with the achieved abscopal effect are summarized in Table 3.

Our case report presents the successes achieved with immunotherapy and cryotherapy in combination. The induction of a systemic response with an abscopal effect by cryotherapy is rare even though the immune system’s stimulation is similar to radiotherapy or oncolytic viruses [18,72]. The combination of costly immunotherapy with more affordable methods could beneficially increase therapeutic outcomes at least in some patients. However, we are recently lacking reliable biomarkers predicting such a favorable response.

## Figures and Tables

**Table 1:** Serological and blood count markers used and their laboratory standard deviations, and critical values (Institute of Medical Biochemistry and Laboratory Diagnostics, General University Hospital and First Faculty of Medicine, Charles University, Prague, Czech Republic).

Serological Analysis	Lower Value Limit	Uper Value Limit	Critical Value Limit	Units
S100B	0.00	0.11	1	g/L
LDH	2.20	3.80	15.00	μkat/L
CRP	0.00	5.50	100.00	mg/L
Blood count				
Neutrophils abs	2.00	7.00	50.00	10^9^/L
Lymphocytes abs	0.80	4.00	7.20	10^9^/L

**Table 2:** Antibodies used for immunohistochemical analysis.

Primary Antibody (Clone No.)	Supplier (Location)
MiTF (Clone D5), MoMoAb, 1:100	Dako, Agilent Technologies, Inc. (Santa Clara, CA, USA)
HMB45 (Clone HMB-45), MoMoAb, 1:100	
MiTF (Clone D5), MoMoAb, 1:100	
MELAN A (A103), MoMoAb, 1:100	
CD68 (M0814), MoMoAb, 1:100	
CD45 (SAB4502541) RaMoAb, 1:200	Sigma-Aldrich, Prague, Czech Republic
CD8 (Clone SP239), RaMoAb, 1:100	
**Secondary Antibody (Clone No.)**	**Supplier (Location)**
N-Histofine Simple Stain MAX PO (414152F)	EXBIO Prague s.r.o. (Prague, Czech Republic)
**Chromogen**	**Supplier (Location)**
DAB (3,3′-Diaminobenzidine)	Dako, Agilent Technologies, Inc. (Santa Clara, CA, USA)
MoMoAb, Mouse Monoclonal Antibody; RaMoAb, Rabbit Monoclonal Antibody; RaPoAb, Rabbit Polyclonal Antibody	

**Table 3:** Key works that have been discussed and where the abscopal effect has been observed were summarized.

Inductor	Clinical/Experimental	Checkpoint Inhibitor	Reference
Radiotherapy	Clinical	Anti-CTLA-4	[19,32,33]
Radiotherapy	Experimental (mice)	Anti-PD1	[35]
Radiotherapy	Clinical	Anti-PD1	[55]
Thermal ablation	Experimental	Anti-CTLA4/Anti-PD1	[60]
T-VEC	Clinical	Not used/Anti-PD1	[28,71]

## Data Availability

The data are available in author’s archive.

## Abbreviations

Aside from official gene names, the following abbreviations were used in-text and legends:

APCs	Antigen-presenting cells
ATP	Adenosine triphosphate
CD4	Cluster of differentiation 4
CD8	Cluster of differentiation 8
CD28	Cluster of Differentiation 28
CD 40	Cluster of differentiation 40
CD48	Cluster of Differentiation 48 (B-lymphocyte activation marker)
CD68	Cluster of Differentiation 68
CD 80	Cluster of differentiation 80
CRP	C-reactive protein
CT	Computed Tomography
CTLA-4	Cytotoxic T-lymphocyte antigen 4, CD152
DAMPs	Damage-associated molecular patterns
DAB	3:3′-Diaminobenzidine
DCs	Dendritic Cells
Gy	Gray (unit)
H&E	Hematoxylin eosin staining
HMGB1	HMGB1
HRP	horseradish peroxidase
ICAM1	Intercellular Adhesion Molecule 1
ICD	Immunogenic cell death
IFNs	Interferons
IFN-γ:	Interferon gamma
IL	Interleukin
IL-1β	Interleukin 1 beta
IL-18	Interleukin 18
IL-2:	Interleukin 2
IL-6:	Interleukin 6
IL-12β:	Interleukin 12 beta
IPRES	Innate PD-1 RESistance
LDH	Lactate dehydrogenase
MHC	Major histocompatibility complex
NK	Natural killer cell
NRL	Neutrophil to lymphocyte ratio
ORR	Objective response rate
OS	Overall survival
P2 × 7	P2X purinoceptor 7
PBS	Phosphate-buffered saline
PD-1	Programed cell death 1, CD279
PFS	Progression free survival
SD	Stable disease
S100B	S100 calcium-binding protein B
TAMs	Tumor-associated macrophages
TILs	Tumor-infiltrating lymphocytes
TNF-α.	Tumor necrosis factor alfa
VCAM1	Vascular cell adhesion protein 1
